# Improved Rehabilitation Outcomes for Persons With and Without Problematic Substance Use After 2 Years With Assertive Community Treatment—A Prospective Study of Patients With Severe Mental Illness in 12 Norwegian ACT Teams

**DOI:** 10.3389/fpsyt.2020.607071

**Published:** 2020-12-23

**Authors:** Hanne Clausen, Torleif Ruud, Sigrun Odden, Jūratė Šaltytė Benth, Kristin Sverdvik Heiervang, Hanne Kilen Stuen, Anne Landheim

**Affiliations:** ^1^Department of Research and Development, Division of Mental Health Services, Akerhus University Hospital, Lørenskog, Norway; ^2^Norwegian National Advisory Unit on Concurrent Substance Abuse and Mental Health Disorders, Innlandet Hospital Trust, Brumunddal, Norway; ^3^Institute of Clinical Medicine, University of Oslo, Oslo, Norway; ^4^Health Services Research Unit, Akershus University Hospital, Lørenskog, Norway; ^5^Centre for Medical Ethics, Faculty of Medicine, University of Oslo, Oslo, Norway; ^6^Faculty of Social and Health Sciences, Inland Norway University of Applied Sciences, Elverum, Norway

**Keywords:** severe mental illness, co-occurring substance use, assertive community treatment, housing, activity, psychiatric symptom, functioning, quality of life

## Abstract

**Background:** Persons with severe mental illness often face difficulties in accessing and receiving adequate services enabling them to live independently. Many have co-occurring substance use problems that increase the risk of adverse outcomes. Community-based service models have been implemented around the world, including assertive community treatment (ACT), but the knowledge of rehabilitation outcomes in different subgroups is limited. We aimed to explore rehabilitation outcomes among patients suffering severe mental illness with and without substance use problems who had received ACT services for at least 2 years. Additionally, we compared differences in changes between the two groups.

**Methods:** A total of 142 patients who received services for 2 years from the first 12 Norwegian ACT teams were included. Eighty-four (59%) had problematic substance use, while 58 (41%) did not. Data regarding housing, activity, symptoms, functioning, and subjective quality of life were collected upon enrollment into ACT and at 2 years of follow-up. Clinician-rated scales and self-report questionnaires were used. Changes within the two groups and differences in change between the groups were assessed using generalized linear mixed models.

**Results:** Both groups were more likely to have good housing, higher level of functioning, and less anxiety and depressive symptoms after 2 years. The odds of good housing among participants with problematic substance use increased only after adjusting for age and gender. Participants with problematic substance use had less severe symptoms, particularly negative and manic symptoms, while participants without problematic substance use reported improved satisfaction with life in general. Neither group experienced a change in having a meaningful daily activity, positive symptoms, practical and social functioning, or subjective quality of life. The reduction of manic symptoms in the substance use group was the only difference between the groups.

**Conclusion:** After 2 years, patients with and without problematic substance use experienced improvements in several important domains. Furthermore, the improvements were similar in both groups for most outcomes. This may suggest that ACT has a place in the continued effort toward integrated and comprehensive community services empowering patients with severe mental illness to achieve and sustain an independent life, including marginalized groups with severe substance use.

## Introduction

Persons with severe and persistent mental illness often struggle with having their needs met by the service system, and health care services often face difficulties in reaching, engaging, and providing services that enable them to live their lives independently in the community. Co-occurring substance use disorders are frequent in this population (1–5), and they increase the risk of adverse outcomes, including relapse or worsening of psychiatric symptoms (6–8), impaired functioning (5, 6), housing instability (9), and lower quality of life (7, 8). Over the past decades, many countries have implemented integrated models of mental health care to improve services for persons with severe mental illness and complex needs (10), including co-occurring substance use problems.

The overarching goal of mental health rehabilitation services for persons with severe and complex mental health conditions is to provide high-quality services that promote recovery and that are based on the patients' needs, wishes, and active participation (11, 12). Appropriate interventions that target a range of factors on an individual level and are provided in the community are needed to improve rehabilitation outcomes and to promote recovery for this group (13). Although the shift from institution-based and fragmented services to integrated, community-based services started decades ago, the services are still insufficient, leading both the World Health Organization (WHO) (14) and the United Nations (UN) (15) to recently emphasize the continuous need to “invest in psychosocial services that are integrated into primary care and community services to empower users and respect their autonomy” (15). A fragmented service system and traditional office-based mental health care, even when localized in the community, may present obstacles to delivering comprehensive and coordinated services, as was the situation in Norway in the early 2000s (16).

One multidisciplinary, team-based, and intensive service delivery program with a strong focus on providing services to improve their patients' abilities to achieve and sustain an independent life in the community is the assertive community treatment (ACT) model (17). ACT teams target persons with severe mental illness, including persons with co-occurring substance use disorders (18, 19), who have complex needs and difficulties in engaging with standard care. These teams use multiple strategies to reach and keep in contact with their patients. High-fidelity teams provide psychosocial and outreach services that are based on the patients' wishes and needs, and the services are evidence-based, individually tailored, and recovery-oriented (20). The implementation of ACT teams in the Norwegian health care system started in 2007 and was included in a white paper in 2009 (21). A main aim was to integrate different health and welfare services to improve treatment for persons with severe mental illness in need of comprehensive and long-term care (16), including persons with co-occurring substance use problems.

Integrated multiple interventions have been found superior over single interventions in improving outcomes for persons with schizophrenia (13). Among persons with co-occurring substance use disorders, they have shown to improve drop out of services, symptom severity, substance use, and housing conditions (22). High-fidelity ACT teams seem to be somewhat better in improving substance use problems and reducing high use of inpatient care than low-fidelity teams, but ACT has not been shown to significantly improve other outcomes over non-intensive or standard care (22). The lack of effect may partly be explained by the heterogeneity of the included studies (19, 22, 23), or that there are too many organizational similarities between the control services and the experimental (ACT) services (23, 24). However, the included studies did not compare outcomes between different subgroups. A recent study comparing outcomes between persons with co-occurring substance dependence, co-occurring substance abuse, or no substance use found that all groups experienced improved outcomes (25). Nevertheless, patients with co-occurring dependence showed less improvements in psychiatric symptoms, level of functioning, and quality of life than patients with substance abuse or patients without substance use problems (25). Further studies are needed to increase our knowledge of rehabilitation outcomes among different subgroups of patients with severe mental illness who receive integrated and community-based services such as ACT, including those with co-occurring substance use disorders.

In the current study, we aimed to explore if outcomes associated with rehabilitation changed for patients both with and without problematic substance use after 2 years with ACT. The outcomes we included were housing situation, meaningful daily activity, severity of psychiatric symptoms, level of functioning, and subjective quality of life.

Our research questions were as follows:

1. Do patients with and without problematic substance use experience changes in rehabilitation outcomes after 2 years of ACT follow-up compared to the situation upon enrollment into the teams?
2. Are any changes in rehabilitation outcomes different for patients with problematic substance use compared to patients without problematic substance use?

## Methods

### Design

This study is part of the research-based evaluation of ACT teams in Norway. It has a prospective cohort design and includes data from patients upon their enrollment into ACT and after 2 years of follow-up.

### Setting

From 2009 until 2011, 12 ACT teams were established throughout Norway in both rural and urban areas. Details regarding the setting and differences between the teams, including fidelity to the ACT model, have been published earlier, but relevant information is repeated here: Fidelity was measured using the Tool for Assertive Community Treatment fidelity scale (TMACT) (26). The mean fidelity scores at 12 months ranged from 2.7 to 3.7, indicating low to moderate fidelity to the ACT model. At 30 months, the scores ranged from 3.1 to 4.1, indicating moderate to high fidelity. Substance abuse specialist was present in 11 teams at 12 and 30 months' fidelity evaluation. The mean fidelity scores on the five subscales relating to substance abuse specialist and Integrated Dual Disorder Treatment (IDDT) showed moderate to high fidelity. However, the scores on the different items showed large variations between teams (scores ranged 1–5, indicating none to full implementation) (27, 28).

### Recruitment and Participants

The recruitment process and the characteristics of the participants, including the classification of problematic substance use, have been described in detail elsewhere (28). However, a brief description is provided: The ACT teams included 338 patients during their first year of operation, and 178 (53%) gave written informed consent to participate in our study. After 2 years of follow-up, 16 of the 178 patients were discharged from the teams, five patients had died, and for 12 participants, no data were shared with the research group despite written informed consent. This left a total of 142 (42%) who had received ACT services for at least 2 years and provided the research group with data from both enrollment and after 2 years of follow-up. Compared to the nonparticipants (*n* = 196, 58%), fewer participants had problematic substance use and they had less severe symptoms and better functioning. There were no differences in age, gender, diagnosis of severe mental illness, or people being subject to involuntary outpatient treatment between participants and nonparticipants (28).

Characteristics of the participants have been presented in Norwegian earlier (27), but a short summary is included here. Upon enrollment into the teams, the mean age of the participants was 40 years [standard deviation (SD) 8.7] and the majority was male (*n* = 94, 67%), of Norwegian origin (*n* = 114, 84%), unmarried (*n* = 106, 75%), and more than half was living alone (*n* = 86, 61%). Seventeen percent (*n* = 33) were either living in institutions or homeless. Almost all participants had a severe mental illness according to the criteria of the International Statistical Classification of Diseases and Related Health Problems (ICD), 10th revision (29), where schizophrenia-spectrum disorders were the most common (F20–29 *n* = 115, 87%) and a few participants had bipolar disorder (F31) (*n* = 9, 7%). Approximately one third was under involuntary outpatient treatment (*n* = 51, 36%). Of the 142 participants, 84 (59%) were classified as having problematic substance use upon enrollment into the ACT teams, while 58 (41%) were not. Further details regarding the classification and characteristics of the two groups have been published in the previously mentioned paper (28), but the categorization and the main differences between the groups are repeated here. Classification of problematic substance use was based on the participants' self-report of alcohol and substance use [the Alcohol Use Disorder Identification Test (AUDIT) and the Drug Use Disorder Identification Test (DUDIT) scales; for more information, see the following paragraph–Measures]. A total of 72 participants (51%) scored above cutoff on one or both scales. For participants who scored below cutoff or who had not completed the AUDIT or the DUDIT, we included the clinician-rated scores on the Alcohol Use Scale (AUS) and the Drug Use Scale (DUS) (for more information, see the following paragraph–Measures). When the score was 3 or higher on one or both scales, the participants were assigned to the “problematic substance use” group (28).

The participants with problematic substance use were more likely to be of Norwegian origin than the participants without problematic substance use; they had a lower level of education; they were more often subject to involuntary outpatient treatment; they had more severe psychiatric symptoms, particularly manic symptoms; and they had a lower level of everyday functioning. There were no differences between the groups regarding gender, age, employment status, living situation, or level of global functioning (GAF-F) upon enrollment. After 2 years of follow-up by ACT, six (7%) of the 84 participants who were classified as having problematic substance use upon enrollment into ACT no longer meet the criteria. We also found that four (7%) of the 58 participants who did not meet the criteria upon enrollment were classified as having problematic substance use 2 years after.

### Measures

#### Clinician-Rated Instruments

Sociodemographic data were collected by the teams using a registration form on their life situation and health that included questions regarding the patients' housing situation and occupational/educational activities. The ACT teams categorized the participants' housing situation as “Very poor” = 0, “Poor” = 1, “Neither poor nor good” = 2, “Good” = 3, or “Very good” = 4 based on their knowledge and observations. Due to few participants in some categories, we dichotomized the variable into “Poor” (including 0, 1, and 2) and “Good” (including 3 and 4) for the analyses.

The ACT teams also assessed the patients' activity situation, including competitive work, supported or sheltered work, studying, unemployment, admitted or incarcerated, or other activities during the last 4 weeks before enrollment and during the last 4 weeks before the 2-year assessment. We dichotomized the variable into “Meaningful daily activity–Yes/No,” where “Yes” included competitive work, sheltered/supported work, and studying, while “No” included unemployment, admissions in institution, or incarceration. This was done due to a very low number of participants in several categories.

Psychiatric symptoms were assessed by the ACT teams using the Brief Psychiatric Rating Scale–Expanded version (BPRS-E) (30). This is a 24-item rating scale, and each item is given a score from 1 (not present) to 7 (extremely severe). The 24 items give four symptom-dimensions, including positive symptoms (grandiosity, suspiciousness, hallucinations, unusual thought content, bizarre behavior, disorientation, and conceptual disorganization), negative symptoms (blunted affect, emotional withdrawal, and motor retardation), agitation mania (tension, uncooperativeness, excitement, distractibility, motor hyperactivity, and mannerism and posturing), and anxiety and depressive symptoms (anxiety, depression, suicidality, and guilt) (31). The reliability of the BPRS in our study was found to be moderate for the BPRS total score [intraclass correlation coefficient (ICC) 0.54]. It was also moderate for the symptom-dimensions positive symptoms (ICC 0.71) and agitation mania (ICC 0.72), while it was poor for negative symptoms (ICC 0.44) and good for anxiety and depressive symptoms (ICC 0.78) (27).

The level of functioning was assessed using the Global Assessment of Functioning (GAF) Scale (32) and the revised version of the Practical and Social Functioning (PSF) scale (33). We used the function scale from the split version of the GAF (GAF-F) (34, 35), where the level of functioning (GAF-F) and the severity of symptoms (GAF-S) are scored separately. The GAF-F scale ranges from 0 to 100, and higher scores indicate better functioning. The PSF revised is a 32-item clinician-rated questionnaire giving eight subscales. Each item is given the score 0 (not able to perform), 1 (partly able to perform), or 2 (fully able to perform). Each subscale comprises four items, with scores ranging from 0 to 8. The PSF mean score is based on the scores on these eight subscales and ranges from 0 to 8. Each subscale is a separate factor with good internal consistency (Cronbach's alpha between 0.735 and 0.903) and acceptable face validity (Personal communication, Ruud, 2014).

The clinicians assessed the participants' problems related to substance use with the AUS (36) and the DUS (37). Both scales are rated on a 5-point scale from 1 (abstinent) to 5 (dependence with institutionalization).

#### Self-Report Questionnaires

The participants also reported their use of alcohol and other substances with the AUDIT (38) and the DUDIT (39). Both questionnaires assess problematic use. The AUDIT comprises 10 items with a total score ranging from 0 to 40. The DUDIT comprises 11 items, and the total score ranges from 0 to 44. Higher scores indicate more severe problems on both questionnaires.

Quality of life was assessed using the Manchester Short Assessment of Quality of Life (MANSA) (40). The MANSA is a self-report questionnaire comprising 11 life domains and one overall question regarding “General life satisfaction.” Each of these 12 items are given a score from 1 (couldn't be worse) to 7 (couldn't be better), and the MANSA mean score ranges from 1.0 to 7.0.

### Data Collection

Sociodemographic and clinical data were collected upon enrollment into the ACT teams and after 2 years of follow-up. Baseline data were collected from December 2009 to February 2012, while 2 years of follow-up data were collected from December 2011 to February 2014. Onset of data collection depended on when the team was established and when the participants enrolled.

Data regarding the participants' life situation and health, psychiatric diagnosis and substance use (AUS and DUS), severity of symptoms (BPRS-E), and level of functioning (GAF-F, PSF) were obtained using clinician-rated instruments. The ACT team members completed the forms based on information from observations, interviews with participants, interviews with relatives and professionals from other services, and from electronic medical records. Information regarding the frequency and the severity of substance use (AUDIT and DUDIT) and the participants' subjective quality of life (MANSA) was obtained from self-report questionnaires that the participants completed alone or together with an ACT team member.

### Statistical Analyses

Sociodemographic and clinical characteristics upon enrollment and after 2 years of follow-up were described as frequencies and percentages for dichotomous and categorical variables and as means and standard deviations (SD) for continuous variables.

Generalized linear mixed models were used to assess changes in outcomes within the two groups (with and without problematic substance use) and differences in changes between the two groups for dichotomous variables. Linear mixed models were estimated to assess changes in outcomes within the groups and differences in changes between the two groups for continuous variables. The models contained fixed effects for the two time points (enrollment vs. follow-up), for substance use status (Y/N), and for the interaction between these two. We included random intercepts for teams to correctly adjust the estimates for possible within-team correlations. We present the results for dichotomous variables as within- and between-group odds ratios (ORs) with 95% confidence interval (CI) and *p*-values. The results for continuous variables are presented as mean within-group changes and between-group differences in change with corresponding 95% CI and *p*-values. All models were further adjusted for age and gender.

As reported in the Recruitment and Participants section, six (7%) of the 84 participants who were classified as having problematic substance use upon enrollment into ACT no longer meet the criteria after 2 years of follow-up, and four (7%) of the 58 participants who did not meet the criteria upon enrollment were classified as having problematic substance use 2 years after. To test if our results were influenced by their status, we performed sensitivity analyses by excluding these 10 participants and reestimating the models above after.

Missing values for the PSF scale (*n* = 14, 0.3% of cases upon enrollment and *n* = 10, 0.2% of cases at 2 years of follow-up), the MANSA (*n* = 12, 0.5% of cases upon enrollment and *n* = 49, 2.2% at 2 years of follow-up), and the BPRS scale (*n* = 0 cases upon enrollment, *n* = 6, 0.2% at 2 years of follow-up) were imputed by generating the empirical distribution for each item and drawing a random number from that distribution to replace the missing value. The process was repeated until all missing values were imputed. The GAF-F scores were close to normally distributed, and missing values (*n* = 4, 2.8% of cases upon enrollment and *n* = 1, 0.7% of cases at 2 years of follow-up) were therefore imputed by drawing a random number from the corresponding normal distribution.

Missing values on demographic variables were not imputed.

The statistical analyses were performed by Statistical Package for the Social Sciences (SPSS) version 25 and SAS software version 9.4. All tests were two-sided, and the results with *p*-values below 0.05 were considered statistically significant. No adjustment for multiple testing was implemented.

### Ethics

This study is a part of the national research-based evaluation of ACT teams in Norway, which has been approved by the Regional Committee for Medical and Health Research Ethics, region South East (ID: 2010/1196a), and by the Data Protection Officer at Innlandet Hospital Trust, Norway.

## Results

Results from the descriptive analyses of the rehabilitation outcomes among participants with and without problematic substance use are reported in Table 1. Results from the regression analyses regarding changes in outcomes within each group and the difference in change between the groups are presented in this section. A short summary of the results from the sensitivity analyses is also provided.

**Table 1:** Characteristics upon enrollment and at 2 years of follow-up.

	**Upon enrollment**		**At 2 years of follow-up**	
**Good housing situation**	***N***	**%**	***N***	**%**
Substance use group	41	50.0	53	64.6
Non-substance use group	35	62.5	45	80.4
**Meaningful daily activity**				
Substance use group	7	8.3	10	11.9
Non-substance use group	8	14.0	12	21.1
BPRS mean score^*^	**Mean**	**SD**	**Mean**	**SD**
Substance use group	*2.60*	*0.86*	2.38	0.81
Non-substance use group	*2.24*	*0.66*	2.09	0.69
BPRS positive symptoms^*^				
Substance use group	*2.65*	*1.34*	2.57	1.31
Non-substance use group	*2.23*	*1.14*	2.14	1.12
BPRS negative symptoms^*^				
Substance use group	*2.43*	*1.14*	2.07	0.96
Non-substance use group	*2.59*	*1.18*	2.34	0.99
BPRS agitation mania^*^				
Substance use group	*2.42*	*1.19*	2.07	0.96
Non-substance use group	*1.78*	*0.77*	1.78	0.78
BPRS anxiety/depressive symptoms^*^				
Substance use group	*2.77*	*0.95*	2.43	0.98
Non-substance use group	*2.63*	*1.10*	2.33	1.05
GAF-Function^*^				
Substance use group	*38.9*	*8.1*	42.6	10.3
Non-substance use group	*40.8*	*8.6*	44.7	12.4
PSF score^*^				
Substance use group	*4.05*	*1.50*	4.32	1.51
Non-substance use group	*4.63*	*1.62*	4.70	1.24
**MANSA mean score**				
Substance use group	4.27	1.09	4.37	0.90
Non-substance use group	4.48	0.90	4.60	0.84
**MANSA life in general**				
Substance use group	4.15	1.72	4.32	1.33
Non-substance use group	4.10	1.55	4.51	1.39

* *Data from enrollment (in italic) have been published in a previous paper (28)*.

*BPRS scores range from 1 to 7, with higher scores indicating more severe symptoms*.

*PSF scores range from 0 to 8, with higher scores indicating better functioning*.

*MANSA scores range from 1 to 7, with higher scores indicating more satisfaction*.

*BPRS, Brief Psychiatric Rating Scale; GAF, Global Assessment of Functioning Scale; MANSA, Manchester Short Assessment of Quality of Life; PSF, Practical and Social Functioning*.

### Housing Situation

The general linear mixed models showed that the odds of having a good housing situation increased significantly for participants with problematic substance use only when adjusting for age and gender (Table 2). For participants without problematic substance use, the odds increased both in the unadjusted and the adjusted model (Table 2).

**Table 2:** Within-group changes from ACT enrollment to 2 years of follow-up—Dichotomous variables.

	**Unadjusted analysis**			Adjusted analysis^*^		
	**OR**	**95% CI**	***P*-value**	**OR**	**95% CI**	***p* value**
**Good housing situation**						
Non-substance use group	2.52	1.07–5.94	**0.035**	2.63	1.10–6.28	**0.030**
Substance use group	1.78	0.95–3.32	0.073	1.92	1.01–3.65	**0.047**
**Meaningful daily activity**						
Non-substance use group	1.52	0.56–4.15	0.414	1.62	0.54–4.88	0.395
Substance use group	1.52	0.53–4.32	0.433	1.34	0.46–3.91	0.589

* *Adjusted for age and gender. The bold values highlight significant p-values (below 0.05)*.

When comparing the change between the two groups, we found that the participants without problematic substance use did not have a significantly greater increase in odds of having a good housing situation compared to the group with problematic substance use (Table 3). Both the unadjusted and the adjusted models showed the same result.

**Table 3:** Between-group changes from enrollment to 2 years of follow-up—Dichotomous variables.

**Dichotomous variables**	**Unadjusted analysis**			Adjusted analysis^*^		
	**OR**	**95% CI**	**p value**	**OR**	**95% CI**	**p value**
Good housing situation	1.49	0.49–4.10	0.515	1.37	0.47–4.04	0.567
Meaningful daily activity	1.00	0.24–4.26	0.999	1.20	0.26–5.60	0.812

* *Adjusted for age and gender*.

### Meaningful Daily Activity

We found that the odds of having a meaningful daily activity after 2 years with ACT did not change significantly within the groups (Table 2), and there was no significant difference in change between the groups (Table 3) in either of the models (unadjusted and adjusted).

### Psychiatric Symptoms (Brief Psychiatric Rating Scale–Expanded Version Scale)

Both the unadjusted and the adjusted model showed that the participants with problematic substance use had less severe symptoms (BPRS-E mean score), in particular less negative, manic, and anxiety and depressive symptoms (BPRS-E subscales) after 2 years with ACT (Table 4). Participants without problematic substance use experienced a significant reduction of anxiety and depressive symptoms (BPRS-E subscale) after 2 years with ACT in both models (Table 4). Neither group experienced a change in their level of positive symptoms.

**Table 4:** Within-group changes from enrollment to 2 years of follow-up—Continuous variables.

	**Unadjusted analysis**			Adjusted analysis^**^		
**BPRS mean score**	Mean difference^*^	**95% CI**	***P*****-value**	Mean difference^*^	**95% CI**	***p*****-value**
Non-substance use group	0.14	−0.06 to 0.34	0.165	0.12	−0.08 to 0.32	0.251
Substance use group	0.23	0.06 to 0.40	**0.007**	0.20	0.03 to 0.37	**0.020**
**BPRS positive symptoms**						
Non-substance use group	0.07	−0.26 to 0.40	0.665	0.06	−0.27 to 0.40	0.708
Substance use group	0.09	−0.18 to 0.37	0.509	0.04	−0.24 to 0.31	0.784
**BPRS negative symptoms**						
Non-substance use group	0.30	0.00 to 0.60	0.051	0.25	−0.06 to 0.56	0.111
Substance use group	0.32	0.07 to 0.58	**0.011**	0.30	0.05 to 0.56	**0.019**
**BPRS agitation mania**						
Non-substance use group	−0.01	−0.26 to 0.23	0.908	−0.09	−0.34 to 0.15	0.451
Substance use group	0.35	0.15 to 0.56	**0.001**	0.33	0.13 to 0.54	**0.001**
**BPRS anxiety/depressive symptoms**						
Non-substance use group	0.30	0.03 to 0.556	**0.031**	0.31	0.04 to 0.58	**0.028**
Substance use group	0.35	0.12 to 0.57	**0.002**	0.32	0.09 to 0.54	**0.005**
**GAF – Function**						
Non-substance use group	−3.14	−5.56 to 0.64	**0.015**	−2.75	−5.30 to 0.20	**0.036**
Substance use group	−2.76	−4.86 to 0.66	**0.010**	−2.61	−4.73 to 0.49	**0.016**
**PSF score**						
Non-substance use group	−0.08	−0.46 to 0.30	0.682	−0.03	−0.42 to 0.34	0.893
Substance use group	−0.23	−0.55 to 0.09	0.164	−0.21	−0.54 to 0.12	0.221
**MANSA mean score**						
Non-substance use group	−0.16	−0.41 to 0.09	0.206	−0.17	−0.43 to 0.09	0.203
Substance use group	−0.07	−0.29 to 0.16	0.570	−0.07	−0.31 to 0.16	0.541
**MANSA life in general**						
Non-substance use group	−0.51	−0.93 to 0.09	**0.020**	−0.54	−0.98 to 0.10	**0.018**
Substance use group	−0.07	−0.46 to 0.32	0.719	−0.09	−0.49 to 0.31	0.671

* *Positive mean difference indicated a reduction in scores, while negative mean difference indicated an increase in scores*.

** *Adjusted for age and gender*.

*BPRS, Brief Psychiatric Rating Scale; GAF, Global Assessment of Functioning Scale; MANSA, Manchester Short Assessment of Quality of Life; PSF, Practical and Social Functioning. The bold values highlight significant p-values (below 0.05)*.

When comparing the two groups, all analyses showed that the reduction of manic symptoms was significantly greater among participants with problematic substance use than among participants without problematic substance use (Table 5). Changes in other symptoms were not significantly different between the two groups.

**Table 5:** Between-group changes from enrollment to 2 years of follow-up—Continuous variables.

**Continuous variables**	**Unadjusted analysis**			Adjusted analysis^^*^^*^^		
	Mean difference^*^	**95% CI**	***p*-value**	Mean difference^*^	**95% CI**	***p*-value**
BPRS mean score	0.09	−0.18 to 0.35	0.511	0.08	−0.18 to 0.35	0.550
BPRS positive symptoms	0.02	−0.41 to 0.45	0.931	−0.03	−0.46 to 0.41	0.910
BPRS negative symptoms	0.02	−0.37 to 0.42	0.915	0.05	−0.35 to 0.44	0.801
BPRS agitation mania	0.37	0.04 to 0.69	**0.030**	0.43	0.11 to 0.75	**0.010**
BPRS anxiety/depressive symptoms	0.05	−0.30 to 0.40	0.787	0.01	−0.35 to 0.37	0.952
GAF-Function score	0.38	−2.92 to 3.69	0.821	0.14	−3.22 to 3.50	0.935
PSF score	−0.15	−0.65 to 0.36	0.568	−0.18	−0.70 to 0.34	0.503
MANSA mean score	0.09	−0.25 to 0.43	0.589	0.10	−0.26 to 0.45	0.596
MANSA life in general	0.44	−0.15 to 1.02	0.144	0.46	−0.15 to 1.06	0.143

* *Positive mean difference indicated a reduction in scores, while negative mean difference indicated an increase in scores*.

** *Adjusted for age and gender*.

*BPRS, Brief Psychiatric Rating Scale; GAF, Global Assessment of Functioning Scale; MANSA, Manchester Short Assessment of Quality of Life; PSF, Practical and Social Functioning. The bold values highlight significant p-values (below 0.05)*.

### Level of Functioning (Global Assessment of Functioning Scale–Function and Practical and Social Functioning)

The global level of functioning (GAF-F) increased in both groups, while the level of everyday practical and social functioning (PSF) did not change significantly in either group (Table 4). Adjusting for age and gender (Table 4) showed the same results.

Both the unadjusted and the adjusted models showed that the changes in the global level of functioning (GAF-F) and everyday practical and social functioning (PSF) were not significantly different between the two groups (Table 5).

### Subjective Quality of Life (Manchester Short Assessment of Quality of Life)

There was no change in satisfaction with life in general or the MANSA mean score among participants with problematic substance use (Table 4) in both the unadjusted and the adjusted model. Participants without problematic substance use reported higher satisfaction with life in general (MANSA life in general; Table 4) after 2 years, while the MANSA mean score did not change significantly. The results remained the same after adjusting for age and gender.

When comparing the two groups, we found that the change in satisfaction with life in general was not significantly different among participants with compared to participants without problematic substance use (Table 5).

### Summary of the Sensitivity Analyses

In the Recruitment and Participants section, we reported that the status of problematic substance use changed for 10 of the 142 participants. We explored if this change of status influenced the main results and performed unadjusted and adjusted sensitivity analyses where these 10 participants were excluded.

In contrast to the main analyses, the sensitivity analyses showed that the odds of having a good housing situation was not higher after 2 years with ACT for participants with problematic substance use after adjusting for age and gender (OR 1.90, 95% CI 0.98–3.36, *p* = 0.059). We also found that participants without problematic substance use experienced a significant reduction of negative symptoms but only in the unadjusted analyses (BPRS negative symptoms 0.34, 95% CI 0.03–0.65, *p* = 0.033). All other results remained unchanged in the sensitivity analyses.

## Discussion

The main purpose of this study was to explore if patients with and without problematic substance use experienced improvements in outcomes important for achieving and sustaining a meaningful and independent life in the community after 2 years of follow-up by ACT teams. Secondly, we aimed to explore if any changes in the outcomes were different between the two groups.

Our study showed that the odds of having a good living situation were higher at 2 years of follow-up than upon enrollment into ACT for both groups, although it was only significant when adjusting for age and gender among participants with problematic substance use. Further studies should be undertaken to explore the significance of age and gender on living situation among persons with severe mental illness and problematic substance use.

Both groups experienced a reduction of anxiety and depressive symptoms, while only participants with problematic substance use had less severe overall symptoms (BPRS-E mean score) and negative and manic symptoms (BPRS-E subscales) at 2 years of follow-up. The level of positive symptoms did not change for either group. The global level of functioning increased for both groups, but neither group experienced improved everyday practical and social functioning. Participants without problematic substance use reported a higher level of satisfaction with life in general at 2 years of follow-up, while participants with problematic substance use did not report significantly higher satisfaction. Neither group had significantly higher odds of having a meaningful daily activity after 2 years of follow-up by the teams.

Our results show that the only significant difference in change was the greater reduction of manic symptoms among participants with problematic substance use compared to participants without problematic substance use. The significantly greater reduction could partly be explained by the higher level of these symptoms among participants with problematic substance use upon enrollment. Participants without problematic substance use problems had little manic symptoms at both time points. An earlier report from this study showed that the participants reported higher satisfaction with their housing situation after 2 years of follow-up. They were also more satisfied with their employment status (having or not having competitive work) but less satisfied with their physical health after 2 years with ACT. We observed no changes in satisfaction with their mental health (27). It is important to emphasize that neither group experienced a deterioration of any outcomes in this study. This is particularly of interest because the participants with problematic substance use had ongoing and severe substance use problems and fewer involuntary and total inpatient days during ACT follow-up (28).

We expected that participants with problematic substance use would experience less favorable changes than participants without problematic substance use because of the ongoing and severe substance use (28), in line with the study by Ruppelt et al. (25). They found that patients without substance dependence experienced greater improvements in symptoms, level of functioning, and quality of life than patients with substance dependence (25). An interesting point is that their study included and secured IDDT to all participants with substance use disorders during the follow-up period. We found that most but not all patients in need of IDDT received such treatment from the teams in our study (fidelity scores 3.0 at 12 months and 4.0 at 36 months, indicating that 60–89% of the patients in need of IDDT also receive it from the team). Additionally, we found that the availability of a substance use specialist was moderate to high (mean fidelity scores on the related items ranged 3.7–3.8) (41), but there were large variations between the teams (28), as described in *Setting*. Although some of the elements of IDDT were only moderately implemented in the ACT teams in this study, participants with problematic substance use experienced improvements similar to participants without problematic substance use.

However, the lack of differences in changes between the groups in our study is in line with a recent multisite randomized controlled trial by Urbanoski et al. (42). They investigated differences in mental health symptoms, community functioning, and quality of life among patients with and without co-occurring substance use disorder receiving Housing First in ACT or intensive case management compared to patients receiving treatment as usual (42). As in our study, the level of substance use remained high during the study period (43), but both patients with and without substance use problems experienced improvements, and the difference was not greater among patients without substance use problems (42).

A service delivery framework, such as the ACT model, is not by itself sufficient or independent of the rest of the service system. The teams need to collaborate closely with other service providers and agencies. For example, the ACT model emphasizes the importance of rehabilitation services to improve patients' possibilities for an independent living in the community, including systematically providing services to support patients' education and employment, such as the model for Supported Employment & Education (20, 44). In our study, we found that the odds of having a meaningful daily activity did not increase for our participants after 2 years with ACT follow-up. One possible explanation may be the organization of health and welfare services in Norway. Social services typically provide financial, vocational, and educational support, while mental health care traditionally focuses on symptom severity and level of functioning and treatment targeting these. Any collaboration between these services occurs on a random basis, and this fragmentation could be an obstacle for the ACT teams to provide vocational and educational services. Additionally, a model that primarily is based on the person's desire to work as the only eligibility criterion is rather new in the Norwegian care system. Such new models might be more difficult to implement, particularly in the start-up phase, as suggested by Odden et al. (41). Another possible explanation may also be that the teams were in their start-up phase during the first part of the study and had a stronger focus on crisis management and everyday coping and less focus on long-term perspective of the treatment, such as education/employment and illness management. This hypothesis may be supported by the lower level of fidelity on the items for vocational service and illness management at 12 and 36 months of operation [EP1 mean scores (SD) 3.0 ± 1.3–4.0 ± 1.0, EP2 mean scores 3.3 ± 1.6–3.6 ± 1.6, EP3 mean scores 1.0 ± 0.0–1.1 ± 0.3, EP5 mean scores 2.3 ± 0.7–2.5 ± 0.9] (41). However, for services to adapt to a more recovery-oriented approach, it is important that these aspects also get attention and focus during treatment and follow-up. It is an important political and administrative task to make sure that employment, education, and illness management is being brought to the attention of the mental health care providers.

Although we cannot exclude the possibility that the changes in part is caused by regression to the mean, it may also suggest that the ACT model can provide an important framework for delivering evidence-based psychosocial services in the community in line with the recommendations from clinical personnel and researchers (11, 45–47), the WHO (14), and the UN (15). Patients with severe mental illness and complex and comprehensive needs often receive inadequate rehabilitation- and recovery-oriented services and have difficulties in achieving and sustaining an independent life in the community (11, 45, 47).

## Strengths and Limitations

The strength of our study is the inclusion of all the 12 first ACT teams that were established in Norway, and the geographical representation of both urban and rural areas. However, the observational and exploratory design does not allow us to draw causative conclusions. We also did not have a control group. Due to the multiple outcome variables used in our analyses, we increase the risk for false-positive findings. However, as this field is still in need of an increased knowledge regarding rehabilitation for persons with severe mental illness, we have chosen to present our findings as they are, but they should be interpreted with caution. We also must emphasize the possibilities of our study being underpowered as some of the results showed close to a significant change in some outcome after 2 years with ACT. Furthermore, we recruited only 42% of all patients who were enrolled into the ACT teams during their first year. There were more nonparticipants than participants with co-occurring substance use problems, and the participants had less severe symptoms and higher level of functioning than the nonparticipants (28). Therefore, we cannot exclude the possibility that the improvements experienced by our sample could be biased by an underrepresentation of a population with more severe problems. Additionally, this study has a prospective pre–post design with data collection at two time points (enrollment and 24 months), providing information regarding the participants' situation at these two time points. A more frequent data collection time (e.g., every 6 months) would have provided the opportunity to explore fluctuations over time.

By dichotomizing the outcome variables Housing Situation and Meaningful Daily Activity, we reduce the variance, but some categories had small numbers (<5) of participants; hence, the dichotomization was performed to avoid possible type II-errors. Finally, the clinical-reported data were collected by the ACT team members, thus many persons were involved in the data collection and the assessments were not blinded. This could have influenced the reliability of some of the scores.

## Conclusion

Our study shows that not only participants without problematic substance use experience improvements in several areas relevant for rehabilitation after 2 years of ACT services. Also, participants with problematic substance use experienced significant improvements in several areas, and the improvements were similar in both groups. It is important to remember that the ACT population typically is a marginalized group that does not receive adequate and appropriate treatment, particularly those with co-occurring substance use. Our results support the understanding that ACT has a place in the continued effort toward adequate, integrated, and comprehensive community services that provide evidence-based interventions aiming to empower and to help patients with severe mental illness to achieve and to sustain an independent life. And most importantly, this includes persons with severe substance use problems.

## Data Availability Statement

The written consent from the participants does not allow for distribution of the data file to others than the research group that conducted the study. Other researchers that want access to the data may contact the principal investigator (AL), who will answer whether the requested data may be made available in a form that does not violate the written consent from the participants.

## Ethics Statement

The studies involving human participants were reviewed and approved by the Regional Committee for Medical and Health Research Ethics, region South East. The patients/participants provided their written informed consent to participate in this study.

## Author Contributions

TR and AL designed the national evaluation of ACT teams in Norway with significant support from SO, HS, KH, and HC. The research questions were formulated by AL, HC, and JSB. Literature search was performed by HC. Statistical analyses were conducted and interpreted by JSB. HC with substantial support from AL. HC wrote the manuscript, which was revised by AL, TR, JSB, KH, SO, and HS. All authors contributed to the article and approved the submitted version.

## Conflict of Interest

The authors declare that the research was conducted in the absence of any commercial or financial relationships that could be construed as a potential conflict of interest.
